# Behavioural and cognitive mechanisms of Developmental Topographical Disorientation

**DOI:** 10.1038/s41598-020-77759-8

**Published:** 2020-12-01

**Authors:** Ford Burles, Giuseppe Iaria

**Affiliations:** NeuroLab, Department of Psychology, University of Calgary, Calgary, AB Canada

**Keywords:** Neurology, Neurological disorders

## Abstract

Individuals affected by Developmental Topographical Disorientation (DTD) get lost on a daily basis, even in the most familiar of surroundings such as their neighbourhood, the building where they have worked for many years, and, in extreme cases, even in their own homes. Individuals with DTD report a lifelong selective inability to orient despite otherwise well-preserved general cognitive functions, and the absence of any acquired brain injury or neurological condition, with general intelligence reported to be within the normal range. To date, the mechanisms underlying such a selective developmental condition remain unknown. Here, we report the findings of a 10-year-long study investigating the behavioural and cognitive mechanisms of DTD in a large sample of 1211 cases. We describe the demographics, heritability pattern, self-reported and objective spatial abilities, and some personality traits of individuals with DTD as compared to a sample of 1624 healthy controls; importantly, we test the specific hypothesis that the presence of DTD is significantly related to the inability of the individuals to form a mental representation of the spatial surroundings (i.e., a cognitive map). We found that individuals with DTD reported relatively greater levels of neuroticism and negative affect, and rated themselves more poorly on self-report measures of memory and imagery skills related to objects, faces, and places. While performing interactive tasks, as a group, the individuals with DTD performed slightly worse on a scene-based perspective-taking task, and, notably struggled to solve tasks that demand the generation and use of a cognitive map. These novel findings help define the phenotype of DTD, and lay the foundation for future studies of the neurological and genetic mechanisms of this lifelong condition.

## Introduction

Getting lost is a common experience. However, individuals affected by Developmental Topographical Disorientation (DTD) get lost on a daily basis, even in the most familiar of surroundings such as their neighbourhood, the building where they have worked for many years, and, in extreme cases, their own homes^1^. DTD was first reported in a Canadian woman in 2008^2^, a case that was followed by several others around the world^3–9^. Behavioural and neurological evaluations of individuals with DTD reveal a lifelong selective inability to orient despite otherwise well-preserved general cognitive functions, and the absence of any acquired brain injury or neurological condition^3,4,10^, with general intelligence reported to be within the normal range^2,6,7,10^. Therefore, despite getting lost in extremely familiar surroundings, individuals with DTD do not seem to differ in their general cognitive abilities from individuals who have no orientation problems. In all cases, individuals with DTD show the same symptoms^1^: (a) they get lost daily in extremely familiar surroundings, (b) they report experiencing topographical disorientation from childhood, (c) they have no other cognitive complaints (i.e., attentional, perceptual, or memory issues), and (d) they do not report any brain injury or neurological disorders. As suggested by a recent pilot study, DTD appears to aggregate in families, suggesting that there may be genetic factors involved in manifesting this condition^11^.

The neurological mechanisms underlying DTD are currently unknown. The handful of case studies, and a single group study with nine individuals, have not revealed any gross volumetric or structural brain abnormalities^3–5,7^. However, altered functioning of regions of the spatial orientation/navigational network, particularly the posterior cingulate and the retrosplenial cortex, have been most consistently implicated in the few neuroimaging investigations of DTD. For instance, while control subjects display robust retrosplenial and posterior cingulate brain activity when performing tasks requiring the recall of a sequence of landmarks^12^, individuals with DTD perform poorly on such tasks and display no significant posterior cingulate activity^4,13^. Importantly, three recent independent studies in individuals with DTD^3,8,10^ documented perturbations of resting-state functional connectivity between the posterior cingulate cortex, the hippocampal complex, and the prefrontal cortex, among other regions, again with no notable structural abnormalities. Although not conclusive, these findings suggest that DTD may be a disorder best characterized by altered functionality of the brain network critical for spatial orientation and navigation, of which the posterior cingulate cortex is an important hub^14^.

The inability of individuals with DTD to orient in familiar surroundings is related to difficulties in adopting a variety of cognitive strategies known to be useful for orientation^5,6^, including associating directions with landmarks, or following routes based on left–right body turns. In most cases, however, individuals with DTD seem to be unable to form a mental representation of the environment (i.e., a cognitive map) despite extended exposure to their spatial surroundings^5,6^. This specific symptom seems to be in line with a lifelong experience of getting lost—given that cognitive maps are critical to successful orientation in familiar surroundings since, once formed, they allow individuals to reach any target location from anywhere within the environment, and even permit generating alternative, unexplored routes if required^15^. Therefore, the inability of individuals with DTD to form cognitive maps may leave them with a sense of unfamiliarity, frequently triggering topographical disorientation even in the most familiar surroundings. Although this hypothesis seems plausible given the current knowledge on the critical role that cognitive maps play in spatial orientation, to date, the limited number of reported cases of DTD available in the literature is not sufficient to verify this hypothesis and define the phenotype(s) of this developmental condition.

Here, we report the findings of a 10-year-long study aiming at describing the behavioural and cognitive mechanisms of DTD in a large sample of 1211 cases. We describe the demographics, heritability pattern, self-reported and objective spatial ability, and some personality traits of individuals with DTD as compared to a sample of 1624 healthy controls, and importantly, illustrate that the presence of DTD is primarily related to the inability to form spatial cognitive maps.

## Results

### Acronyms

‘BF’ refers to the Bayes Factor for any given variable to be included in a model—larger Bayes Factors indicates stronger evidence of an effect. The odds ratio, ‘OR’, represents the difference ratio of a categorical effect between two groups, and can be interpreted as a measure of effect size. In the present study, odds ratios exceeding one indicate a greater proportion of a characteristic in the DTD sample, whereas ratios smaller than one indicate a greater proportion of a characteristic in the control sample. The mean difference between the DTD sample and control sample on a quantitative variable is indicated by ‘MD’.

### Demographics

The female:male ratio in our DTD sample (a ratio of 5.21:1) was far greater than that in the control sample (a ratio of 1.55:1; χ^2^ = 178.288, *p* = 1.146e−40, OR 3.351), and the participants in the DTD sample (age *M* = 35.66, *SD* = 13.18 years) were on average older than those from the control sample (age *M* = 29.07, *SD* = 12.83 years; Welch *t*_2552.870_ = 13.250, *p* = 8.342e−39). While both the DTD and control samples were predominantly drawn from an urban population (urban:rural ratios of 4.659 and 6.382, respectively), the DTD sample was proportionally constituted by slightly more rural participants (χ^2^ = 9.102, *p* = 0.003, OR 1.370).

### Heritability

Individuals with DTD generally reported more first-degree relatives with orientation difficulties that arose in childhood (χ^2^ = 266.160, *p* = 7.796e−60, OR 2.914; see Table 1), and both control (χ^2^ = 58.850, *p* = 1.702e−14, OR 2.406) and DTD participants (χ^2^ = 122.743, *p* = 1.588e−28, OR 2.494) reported more female than male first-degree relatives with orientation difficulties that arose in childhood.

**Table 1:** First-degree-relative orientation issues contingency table.

	Affected	Unaffected	Odds ratio	χ^2^	*p*
Fathers of controls	26	1152	5.770	75.526	3.607e−18
Fathers of DTDs	103	791			
Mothers of controls	109	1047	3.729	119.896	6.668e−28
Mothers of DTDs	250	644			
Brothers of controls	63	1004	1.940	15.696	7.437e−5
Brothers of DTDs	94	772			
Sisters of controls	101	831	2.420	46.619	8.620e−12
Sisters of DTDs	205	697			
Sons of controls	25	203	1.593	3.233	0.072
Sons of DTDs	52	265			
Daughters of controls	33	212	2.593	18.558	1.648e−5
Daughters of DTDs	88	218			
Overall FDRs of controls	357	4449	2.922	266.160	7.796e−60
Overall FDRs of DTDs	792	3387			

Affected and Unaffected indicate if the family member experienced topographical disorientation, as reported by the proband. Only responses from relationships where the presence of topographical disorientation was explicitly affirmed or denied were included.

*FDR* first-degree relative.

### Behavioural self-report

A smaller proportion of DTD participants (33.691%) as compared to control participants (52.803%) reported playing video games involving walking, navigating, or driving (χ^2^ = 102.512, *p* = 4.288e−24, OR 0.454). However, for those that play such video games, we did not detect significant differences between DTD and control participants in the hours per week currently played (Welch *t*_331.375_ = 0.858 *p* = 0.392), nor the number of years played (Welch *t*_686.441_ =  − 1.489 *p* = 0.137). As expected, DTD participants reported difficulty remembering routes, despite having travelled them multiple times, at far greater rates than control participants (χ^2^ = 937.495, *p* = 6.935e−206, OR 35.519). DTD participants were also far more likely to report getting lost more than once a week while travelling to familiar destinations (without GPS or similar; χ^2^ = 882.238, *p* = 7.129e−194, OR 13.561), as well as getting lost at least once in their own home in the past year (χ^2^ = 38.242, *p* = 6.250e−10, OR 2.520).

### Self-assessment and sense of direction

**Figure 1:**
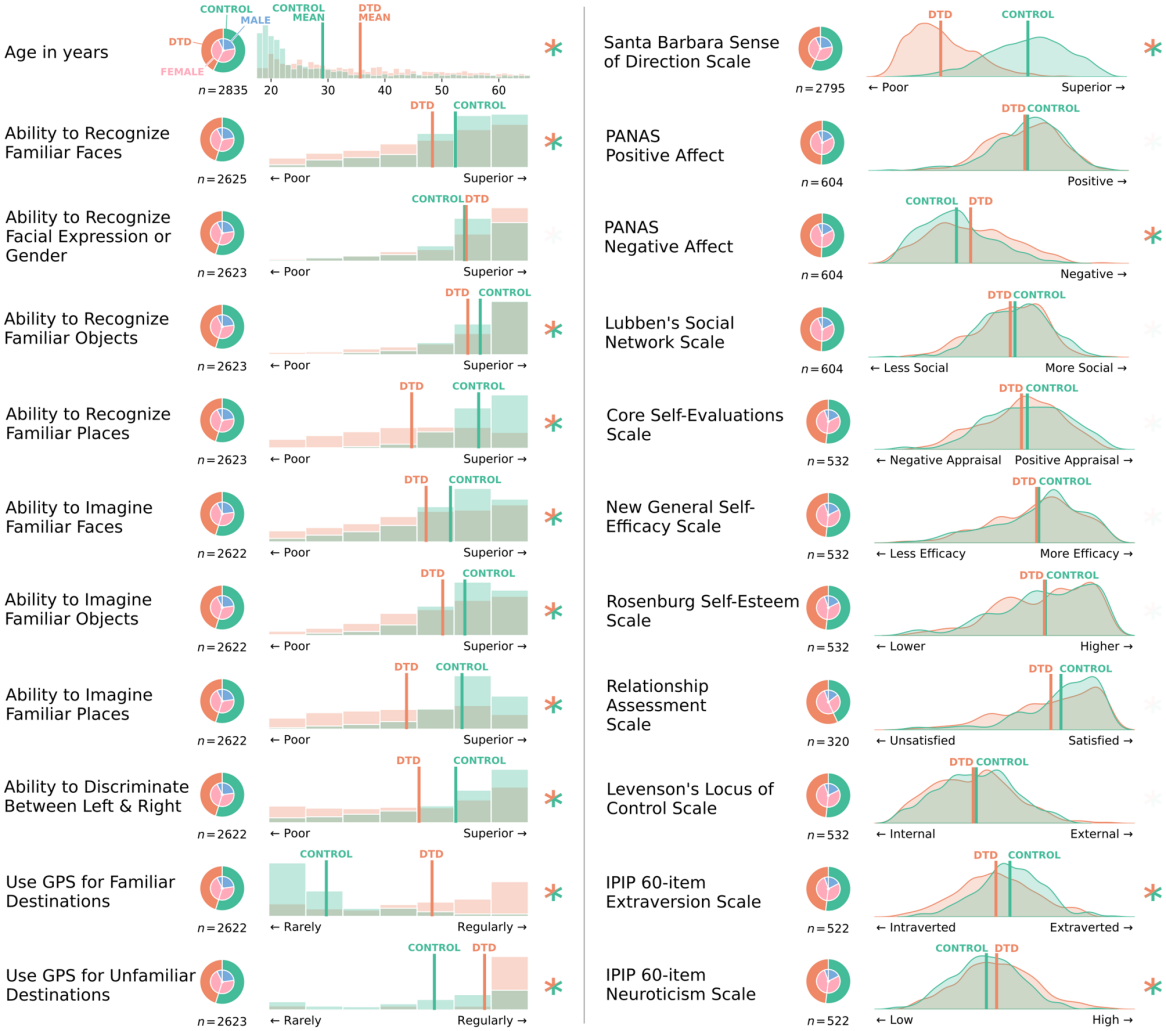
Data from the navigational self-assessment, sense of direction, social, and personality questionnaires from the DTD and control samples. Nested pie charts depict the demographic composition of each variable. Histograms or probability density plots have group means marked with vertical lines. Asterisks indicate evidenced (i.e., Bayes Factors exceeding 10) group differences between those with DTD and controls, with any evidenced effects or interactions including age or sex accounted for. *PANAS* positive and negative affect schedule.

Unsurprisingly, we detected extremely strong evidence that individuals with DTD reported far poorer sense of direction than controls on the Santa Barbara Sense of Direction scale (SBSOD^16^; BF = inf, *MD* = 34.476 [32.646, 36.346]), as well as strong evidence of a DTD by age interaction (DTD*age BF = 188.731) in which older control participants reported better sense of direction (*b* = 0.307, *SE* = 0.029), whereas in older participants with DTD this effect was attenuated (*b* = 0.096, *SE* = 0.027). We detected very strong evidence that individuals with DTD report somewhat worse ability than controls in recognizing familiar faces (BF = 2.275e+11, *MD* = 0.506 [0.330, 0.667]) and objects (BF = 8.071e+9, *MD* = 0.384 [0.243, 0.521]), and particularly strong evidence for worse reports of their ability to recognize familiar places (BF = 3.561e+172, *MD* = 1.810 [1.655, 1.959]), but no difference in their self-reported ability to recognize facial expression (BF = 0.047). Similarly, we detected strong evidence that individuals with DTD reported a poorer capacity to imagine familiar faces (BF = 4.866e+18, *MD* = 0.690 [0.494, 0.877]), objects (BF = 4.584e+22, *MD* = 0.594 [0.448, 0.738]), and places (BF = 3.658e+107, *MD* = 1.498 [1.336, 1.666]). There was also strong evidence that those with DTD report more difficulty discerning left from right (BF = 3.162e+23, *MD* = 0.836 [0.622, 1.040]), and rely on their GPS far more when travelling to familiar (BF = 2.318e+258, *MD* = 2.904 [2.712, 3.093]) and unfamiliar (BF = 1.453e+72, *MD* = 1.45 [1.249, 1.667]) destinations. Control participants (*b* = 0.040, *SE* = 0.004) also reported a greater age-related decrease in GPS use than those with DTD (*b* = 0.015, *SE* = 0.004) when travelling to unfamiliar locations (BF = 3107.978). These data are summarized in Fig. 1.

### Social and personality measures

No sufficiently-evidenced DTD-related effects were detected for positive affect (BFs ≤ 0.511), social engagement (BFs ≤ 0.731), self-efficacy (BFs ≤ 1.277), self-esteem (BFs ≤ 1.711), locus of control (BFs ≤ 0.276), nor relationship satisfaction (BFs ≤ 2.591), excepting the core self-evaluations scale where BFs ≤ 8.776. However, our analyses indicated very strong evidence for individuals with DTD having greater negative affect (BF = 2.525e+6, *MD* = 3.620 [1.999, 5.322]), lower extraversion (BF = 49.203, *MD* = 12.092 [3.196, 22.832]), and greater trait neuroticism (BF = 1341.580, *MD* = 17.480 [6.658, 29.785]). These data are summarized in Fig. 1.

### Interactive tasks

**Figure 2:**
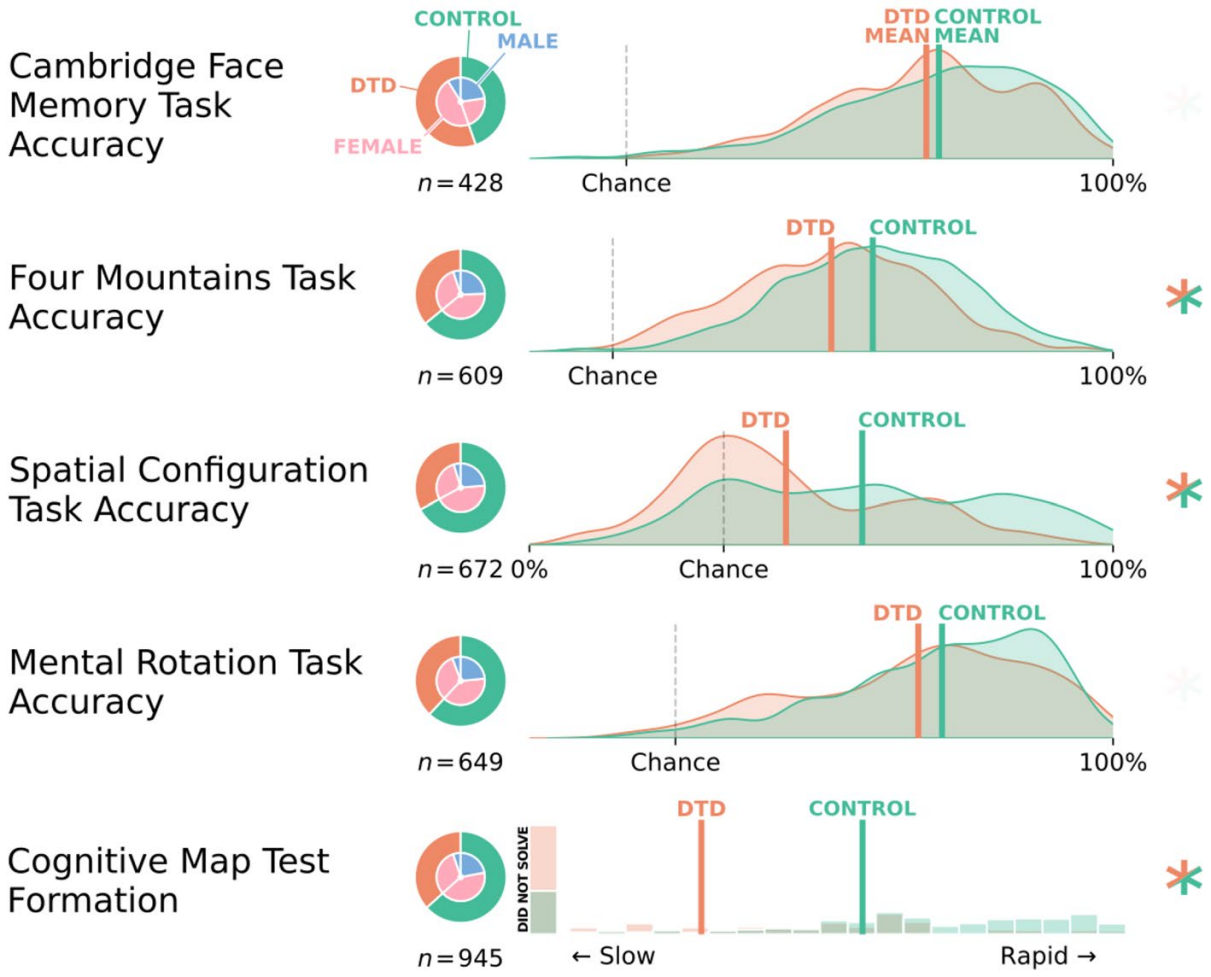
Interactive face processing and spatial assessments. Nested pie charts depict the demographic composition of each variable. Histograms or probability density plots have group means marked with vertical lines. Asterisks indicate evidenced (i.e., Bayes Factors exceeding 10) group differences between the DTD and control group, with any evidenced effects or interactions including age or sex accounted for. The Gettinglost.ca Four Mountains task is a measure of scene memory and spatial perspective taking, the Spatial Configuration Task and Cognitive Map Task are measures of the ability to form a cognitive map of an environment.

Despite the DTD participants reporting a poorer ability to recognize and imagine familiar faces as compared to controls, we did not detect evidence for such a difference in accuracy (BF = 0.247) or reaction time (BF = 0.136) on the Cambridge Face Memory Task^17^. Similarly, we found no evidence of differences in accuracy (BF = 0.169) or reaction time (BF = 0.112) on the Mental Rotation Task. On the other hand, individuals with DTD performed more poorly than controls on our assessment of perspective-taking ability, the Gettinglost.ca Four Mountains Task (BF = 542.562, *MD* = 5.4 [2.0, 9.4]%), and, as expected, performed significantly worse than controls in the two different measures of cognitive map formation, i.e. the Spatial Configuration Task (BF = 2495.242, *MD* = 8.8 [3.0, 14.2]%) and the Cognitive Map Test (BF = 4.178e+16, *MD* = 4.488 [3.155, 5.943] trials to criterion). Correcting for the different chance-level performances in these tasks (i.e., computing the ‘interpretable’ range as the range between the accuracy expected by chance and 100% accuracy), the mean difference between DTD and control groups spanned 22.4% of the interpretable range of the Cognitive Map Test, 13.1% of the interpretable range of the Spatial Configuration Task, and 7.2% of the interpretable range of the Four Mountains Task. These differences are summarized in Fig. 2.

## Discussion

In this report, we have described the various demographic, familial, personality, and spatial measures that appear to be affected in individuals with DTD. This large sample of individuals with DTD all self-reported difficulties orienting and no other cognitive or neurological conditions. Individuals with DTD reported relatively greater levels of neuroticism and negative affect, and rated themselves more poorly on self-report measures of object, face, and place memory and imagery. From our battery of interactive tasks, as a group, the individuals with DTD slightly struggled on a scene-based perspective-taking task (i.e., the Gettinglost.ca Four Mountains Task) and clearly struggled with tasks that demanded they generate and use a mental representation of an environment, i.e., the Spatial Configuration Task and Cognitive Map Test. The performance of individuals with DTD on cognitive-map-based tasks appeared to more saliently differ from that of controls, although none of these tasks provided a clear differentiation between those who reported orientation difficulties and those who did not. These results are largely consistent with our understanding of the importance of cognitive maps for flexible navigation and spatial orientation. An individual unable to form and make use of cognitive maps for navigation is largely restricted to navigating using rigid, route-based strategies^18^, relying on navigational aids such as turn-by-turn GPS navigation systems^19^, or relying on others to wayfind. Individuals with DTD regularly report making use of these compensatory strategies, but they are often situational and do not ameliorate the deficit these individuals experience. Furthermore, the higher reported levels of neuroticism and negative affect in individuals with DTD is likely indicative of an increased level of stress reactivity, with the threat of getting lost a prominent stressor in these individuals’ lives. It is possible that higher levels of neuroticism or negative affect are not core features of DTD, but are more likely to be associated with DTD causing impairments in performing day-to-day navigational activities (potentially via adoption of maladaptive coping strategies^20^, or even agoraphobia^21–23^) and therefore increasing the likelihood of characterizing oneself as having difficulties orienting. In addition to this possibility, neuroticism has been shown to affect the functioning of a wide variety of brain regions, including some implicated in spatial processing e.g., the hippocampus, posterior cingulate, caudate nucleus, and fusiform gyrus^24–26^. It may be the case that neuroticism exists as a risk factor for DTD in a biological sense, in that it may be associated with brain network perturbations in a similar manner to those seen in DTD.

Another interesting feature of our DTD sample was the overwhelming ratio of females to males, at over 5:1. While a substantial portion of this is likely sampling bias, e.g. men less likely to seek help or report difficulties^27–29^, it seems unlikely that this is a complete account of the preponderance of women in our DTD sample. Our sample also reported significantly more female than male relatives with orientation difficulties, although at a more modest ratio of approximately 2.4:1. The larger number of females reporting difficulties orienting may also be partially due to the tendency for males to perform better than females at some spatial tasks^30–33^; the current battery detected evidence of a sex advantage for males in Mental Rotation Task accuracy, Spatial Configuration Task reaction time, and Four Mountains Task accuracy (reported in Supplemental Table S1). These differences may provide a ‘cognitive reserve’, making males more resilient to DTD as they generally can sustain larger perturbations in their spatial abilities before experiencing deficits in their day-to-day functioning. Furthermore, males appear to be noticeably less affected by stress-related impairments to spatial ability as compared to females^34–36^, another mechanism that undoubtedly affects the rates that one would experience getting lost in familiar and unfamiliar places.

Previous research in prosopagnosia, i.e., the impaired ability to recognize familiar faces*,* identified that some of those with face processing issues also experience issues recognizing places or forming cognitive maps^37,38^. This relationship is possibly driven by some degree of overlap between the visual cortex processing faces and scenes^39^, and the close proximity of the Fusiform Face Area (FFA) and Parahippocampal Place Area (PPA)^40^. While our participants with DTD self-reported comparatively poorer capacity to recognize and imagine familiar faces, we did not detect any evidence for differences in performance between individuals with DTD and controls on the Cambridge Face Memory Task. However, it is important to note that our sample would have excluded anyone who reported having prosopagnosia, which may appear co-morbidly at rates greater than expected by chance. In addition, it is possible that individuals with DTD generally have some issues with face processing that are simply not captured by the task we employed^41^, or this is indicative of a bias in evaluating one’s own abilities^42^. The Cambridge Face Memory task employed in the present study utilizes static face stimuli, with peripheral information (e.g., hair, ears) removed. It is possible that assessing the face processing and memory of individuals with DTD with more demanding, ecological stimuli (e.g., short movies^43^, people embedded in spatial and/or social contexts, longer encoding-to-recall delays) would have revealed deficits coherent with their self-report. That is to say that the self-reported face processing difficulties in DTD may be attributable to these complementary processes that are involved in recalling or recognizing people, but short-term face recognition memory specifically does not appear to be grossly impaired in our DTD sample. The generally poorer performance on the Gettinglost.ca Four Mountains Task by individuals with DTD does suggest that there may be a substantial proportion of these individuals experiencing some difficulty processing and remembering complex visual stimuli, but not faces per se. Visual processing of faces and places both are ventral-stream processes, and presumably share a large portion of neural resources, but portions of the cortex that specialize in places (e.g. the PPA) and faces (e.g. the FFA) are distinct enough that these processes are clearly dissociable; intact face and object processing abilities alongside impaired topographical abilities can be seen in some individuals with right posterior cerebral artery infarcts resulting in damage to the parahippocampal or anterior fusiform gyri^44^. While the neurological correlates of performing the four mountains task are not well-described in healthy participants, intact functioning of the hippocampus and parahippocampus appear to be necessary to perform this task^45,46^. Kim et al.'s investigation of J.N., a case of DTD, revealed impaired functional connectivity of a handful of brain regions, including the PPA and hippocampus^3^. Such neurological features may be underlying the difficulties that some of those with DTD experience on the Gettinglost.ca Four Mountains Task.

**Figure 3:**
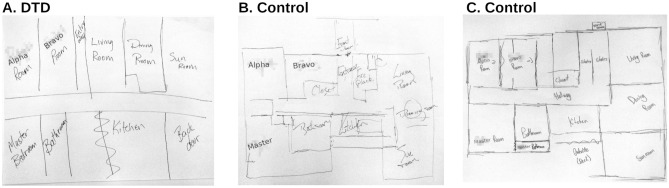
Drawings of the same household by an individual with DTD (**A**), their unaffected spouse (**B**), and their unaffected child (**C**). Notice the lack of metric spatial information present in the drawing in (**A**), in which only the rough order of rooms is reported. Individuals with DTD are often using a verbal strategy when map-sketching: reporting the order of landmarks experienced on a known route, and using that sequence information to produce a rough map.

Moving to a more nuanced understanding of DTD will be difficult. It is clear that spatial orientation and navigation are complex cognitive phenomena that can be supported, compensated for, or perturbed at cognitive^47^, perceptual^48^, or sensory^49^ levels, with the potential for genetic^11^, social-personal^50^ and neurobiological risk factors associated with this condition. There is a need to identify more discrete and objective criteria for identifying DTD, as to-date, we have been largely reliant on self-report measures as the primary diagnostic factors, the present study included. Such self-reports may be exaggerated, or participants may believe they are expected to respond in a certain way (e.g. a social desirability bias), among other possible sources of error^51^. However, that is not to say that subjective reports do not have a place in future investigations in DTD; much like subjective reports of sleep quality appear to capture different variance than that typically captured by objective measures of sleep quality^52,53^, subjective reports of spatial ability (e.g., the Santa Barbara Sense of Direction Scale) are not well explained by any single objective measure of spatial ability^48,54^. Strategy use^47^, acute stressors^55^, and social and technological factors can easily and significantly alter an individual’s subjective wayfinding experiences about that expected from their spatial ability directly. Subjective measures can holistically capture this variability that would be overwhelming to capture in a series of measures designed to capture each factor independently—and assuming all important factors are known and could be captured and combined in this manner. That being said, we feel that there is a need for simple objective tasks that we would reasonably expect those with DTD to be unable to solve, and those without DTD to be able to solve. From our findings on the inability to form cognitive maps, as well as direct experience and reports from those with DTD, the most straightforward task that individuals with DTD struggle with is pointing to unseen landmarks (much like the deficit seen in acquired topographical disorientation resulting from damage to the retrosplenial/posterior cingulate cortex^44^). Most adults, as an example, are able to point in the direction of their home when sitting in their doctors office (typical errors not withstanding^56^), whereas those with DTD often report they are simply guessing when tasked to do this. Another commonly-reported^2,6,7,13^ behaviour in DTD is the spatial-detail-sparse drawings of the layout of their own home or workplace (see Fig. 3). Similarly, we would expect individuals with DTD to not have substantial deficits in the ability to perform lower-level cognitive tasks, such as those measured by the mental rotation task, four mountains task, and face memory tasks, that one would reasonably expect would cause their inability to navigate throughout familiar environments. While these quick assessments can often complement a subjective report of difficulties navigating, we have not yet identified a task with sufficient sensitivity or specificity to diagnose DTD alone. That being said, the present study indicates that of the tasks in our battery, performance on cognitive map-style tasks appear to be the most affected in those individuals struggling with getting lost in their day-to-day lives.

A clearly delineated cognitive-behavioural characterization of DTD will be critical for and complemented by investigations of the neurological correlates of this condition. All individuals with DTD get lost in familiar environments, but the degree to which a given individual struggles with getting lost, as well as other related processes, varies between cases. For instance, ‘Pt1’, an individual with DTD, required 32 min to generate a cognitive map of a simple virtual environment^2^, whereas ‘Dr. WAI’, another case of DTD, performed this task in 9 min, comparable to that of controls^6^. ‘Dr. WAI’ also struggled on measures of mental rotation and other smaller-scale spatial skills, whereas ‘Pt1’ did not. These differences could represent possible taxonomies within DTD^57^, or perhaps simply normal individual variability in spatial skills (and/or compensatory strategies), but regardless, should be reflected in investigations of these individuals’ functional brain architecture. Therefore, inconsistent cognitive-behavioural descriptions of DTD will preclude consistent neurological descriptions. To address this, we suggest that investigations of DTD would greatly benefit from common use of explicit and sensitive assessments of scene processing ability, face processing ability, and mental rotation ability, among other related processes, such as those provided on our online testing platform (gettinglost.ca) and freely available to anyone. It is apparent that there will be individual variability within the population of individuals with DTD^5^. However, we will need to carefully categorize cases that are consistent with the original characterization of DTD—in which the subject’s disorientation appears to be ‘primary’, with no other factor or lower-level process (e.g., visual agnosias) clearly responsible for their navigational deficit—from cases in which another affected cognitive process may be responsible for the individual’s disorientation to some degree^57^, making the subject’s disorientation ‘secondary’ to those deficits. Only from a well-defined symptomology can a cohesive neurological profile be identified, and perhaps effective cognitive-behavioural rehabilitation programs be developed.

## Methods

### Participants

We recruited and tested 4028 participants through an online platform (www.gettinglost.ca) between October 2009 and April 2020. This sample included locally-recruited participants as well as participants who performed the study unsolicited. From these, we identified 1211 participants (65 years old or younger) affected by DTD as indicated by the official inclusion/exclusion criteria for this condition^2^, that is (a) getting lost frequently in extremely familiar surroundings, (b) experiencing the orientation problem consistently from childhood or adolescence (i.e., the stage at which we would expect an individual to begin independently navigating), (c) reporting no other cognitive complaints (i.e., attentional, perceptual, or memory issues), and (d) reporting no brain injury or neurological conditions. In the present study, these criteria were operationalized as follows: (a) participants responded ‘yes’ to the query ‘do you experience difficulties orienting?’, (b) participants responded ‘no’ to the query ‘have these difficulties changed over time?’ and responded to ‘how old were you when you first became aware of or concerned about your lack of orientation skills?’ with an age of less than 20, (c) responded ‘no’ to the query ‘do you have any neurological condition or cognitive disorder including colourblindness?’, and (d) responded ‘no’ to the query ‘Are you aware of having any brain damage, brain malformation, or brain tumour?’. The DTD sample included 1015 females (*M* = 35.79, *SD* = 13.33 years old) and 196 males (*M* = 35.00, *SD* = 12.42 years old), resulting in a female:male ratio of 5.21:1. In addition to the individuals identified as affected by DTD, we identified 1624 healthy individuals (65 years old or younger) who did not report any orientation problems nor, as for the participants with DTD, any cognitive complaints, brain injuries or neurological conditions. The sample of control participants included 986 females (*M* = 27.80, *SD* = 12.44 years old) and 638 males (*M* = 31.01, *SD* = 13.19 years old), a female:male ratio of 1.55:1. The remaining individuals were excluded from the study and all analyses due to either being older than 65 years of age, or not providing clear information allowing us to classify them as either a DTD or control participant. The recruited participants participated in varying assessment batteries, and as such have varying degrees of data completeness. The different questionnaires and interactive tasks that participants completed are detailed below. Although not recorded formally, we estimated that throughout the decade of recruitment and texting one-third of the individuals with DTD reached out to us after completing our assessments, and underwent an unstructured phone interview or email conversation in which we confirmed the lifelong inability to orient in extremely familiar surroundings as reported in our online questionnaires. As we did not objectively assess the presence or absence of other cognitive or neurological conditions in this sample of individuals with DTD, it is possible that some cases are misclassified. The Conjoint Health Research Ethics Board (CHREB) of the University of Calgary approved the study and all research was performed in accordance with its guidelines and regulations. Informed consent was obtained from all participants.

### Demographics and questionnaires

Participants were first asked to complete a demographics questionnaire and answer a series of questions about their navigational and orientation skills and related processes (i.e., the Navigational Self-Assessment) and the Santa Barbara Sense of Direction Scale (SBSOD^16^). Subsequently, participants completed a short questionnaire on the spatial skills of their family members (DTD Family-Heritability; data reported in Table 1). Participants also completed a battery of social and personality measures, consisting of numerous self-report scales including: the Positive and Negative Affect Schedule^58^, social engagement measured by Lubben’s social network scale^59^, the Core Self-Evaluations Scale^60^, the New General Self-Efficacy Scale^61^, the Rosenberg Self-Esteem Scale^62^, and relationship satisfaction measured by the Relationship Assessment Scale^63^, a 5-item version of Levenson’s Locus of Control scale^64^, the IPIP 60-item Extraversion scale, and the IPIP 60-item Neuroticism scale^65, 66^. Sample items from these scales, as well as data completeness are reported in the supplementary materials.

### Interactive tasks

#### The Cambridge face memory test

The Cambridge Face Memory Test^17^ assesses the ability of the individuals to process facial identity information and recognize familiar faces. The task requires participants to memorize six different faces and subsequently identify them among other faces. This specific test was included in the experimental protocol due to some preliminary findings suggesting a possible relationship between the inability to orient and the inability to recognize familiar faces^10,37^, as experienced by individuals affected by prosopagnosia^67^.

#### The mental rotation task

The Shepard and Metzler -style Mental Rotation Task is a well-established measure of the ability to mentally represent and manipulate objects^68^. In each of the 80 trials of this task, participants are required to compare two 3D objects composed of 10 cubes arranged in a non-planar format, placed side-by-side, and identify if the two objects are the same or mirror images of each other. Object pairs are presented at varying degrees of rotational disparity from one another (ranging from − 120° to 120°), about any of the three canonical axes. Participants’ accuracy and reaction times are recorded as metrics of performance.

#### The Gettinglost.ca four mountains task

**Figure 4:**
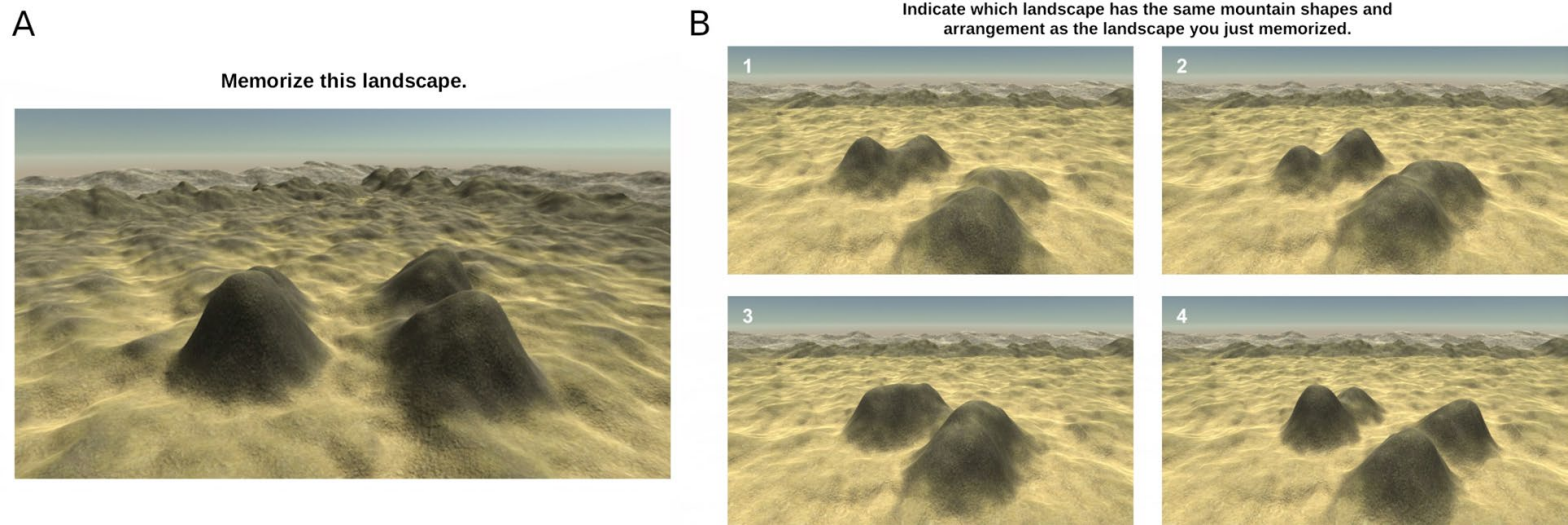
The Gettinglost.ca Four Mountains task. (**A**) Depicts a sample landscape participants would be asked to memorize, and (**B**) depicts a sample set of four response options, in which participants would select the option that shares the same topography as the memorized landscape (in this case, option 4). This figure is a derivative work of materials by Ford Burles, both provided under a CC BY 4.0 license.

The Gettinglost.ca Four Mountains Task (Fig. 4) was designed to assess an individual’s ability to recognize simple scenes from different perspectives, i.e*.* their perspective taking ability. This task was created to mimic Hartley et al. four mountains test^45^, but includes procedurally-generated stimuli and foils (i.e., generated randomly from a handful of parameters), as opposed to Hartley et al. categorically-defined foils (in which each foil has a specific property in which it deviates from the target response), as we felt that this would prevent strategic responding via process of elimination. In each of the 20 trials in this task, participants are first presented with landscape for eight seconds, with four ‘mountains’ in the foreground. Participants are asked to memorize the size, shape, and relative positions of these four mountains. After 2 s of blank screen, participants are presented with four different landscapes: one of these landscapes has the same topography as the studied landscape, but the camera position, lighting angle, and textures will have changed. The remaining three response options depict scenes with disparate topography, but the same camera position, lightning angle, and textures as the target option (see Fig. 4). Participants have an unlimited amount of time to select the response option that shares the same mountain shapes and arrangement with the studied scene; their accuracy and reaction times are recorded as performance metrics.

#### The spatial configuration task

**Figure 5:**
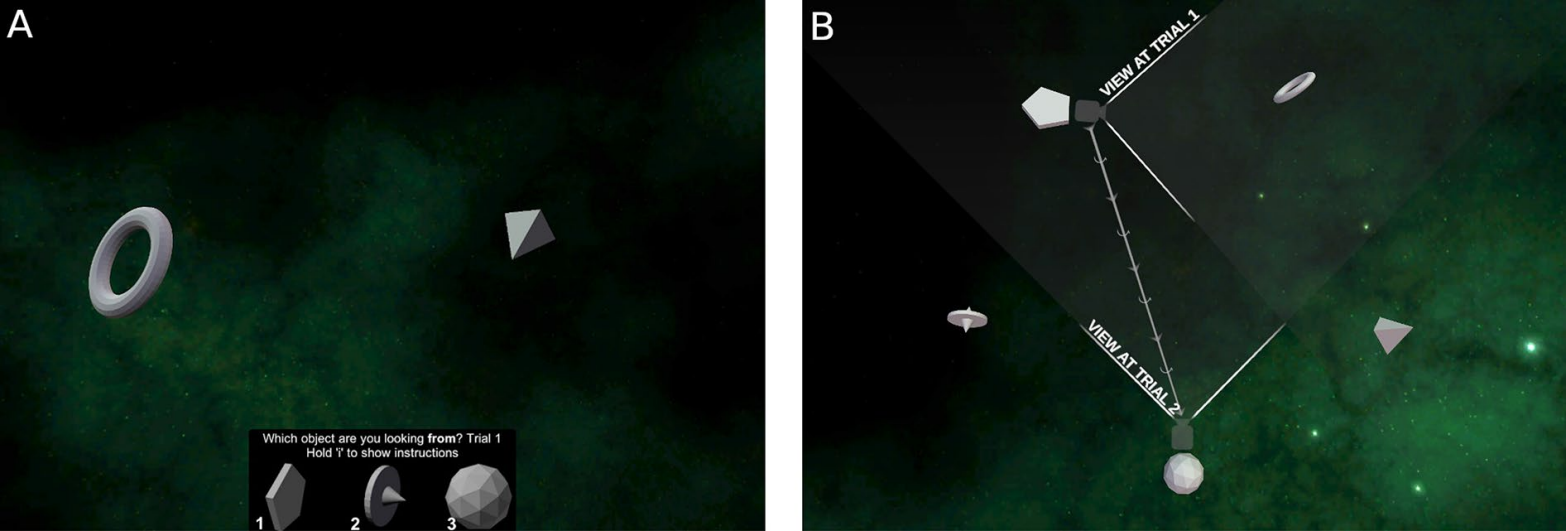
The Spatial Configuration Task. (**A**) Depicts a sample trial from the task, and (**B**) depicts a top-down view of the environment and a short schematic representation of the camera movement and view within a sample pair of trials. Participants never see a top-down view like that presented in (**B**). This figure is a derivative work of materials by Ford Burles, both provided under a CC BY 4.0 license.

The Spatial Configuration Task was designed to assess the ability of the individuals to generate a mental representation, i.e., a cognitive map, of a simple virtual environment^69,70^. This task’s virtual environment is constituted by five simple geometric objects pseudorandomly set in a pentagonal arrangement in a space-like environment. In each of the 60 trials in this task, participants are presented with a view from one unseen object, with two other objects in view (see Fig. 5). Participants have all three unseen objects provided as response options, and have as much time as they require to select the object that they are currently looking from, i.e., the object the camera is currently situated upon. After responding, the camera smoothly translates and rotates to a new object, again with only two objects in view, and a new trial begins. To successfully perform this task, participants are expected to generate a mental representation of the environment over successive trials, and infer the camera’s location from the depicted viewpoint at each trial. Accuracy and reaction times are recorded as performance metrics.

#### The cognitive map test

**Figure 6:**
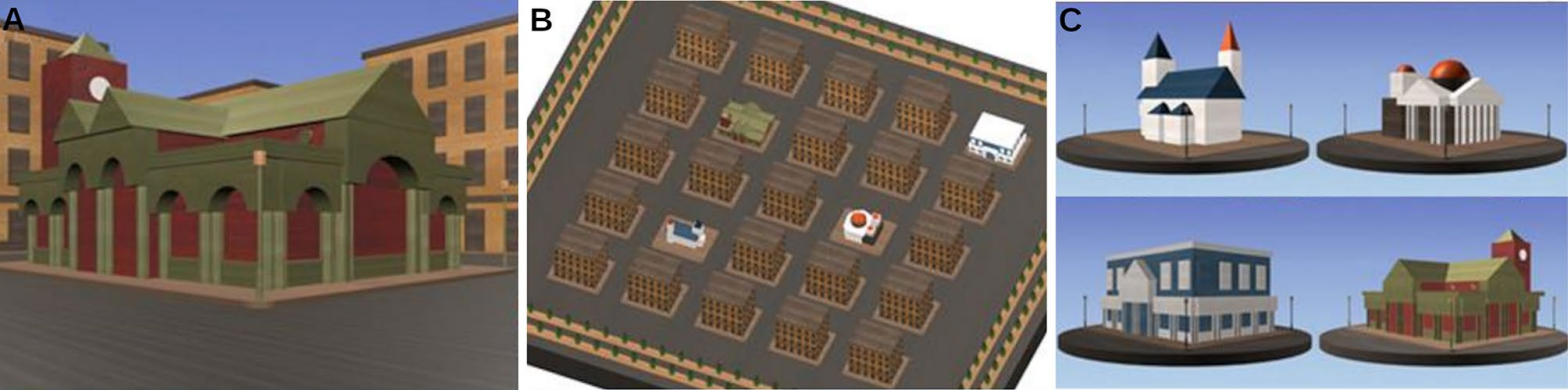
The Cognitive Map Test. (**A**) Depicts a sample view of a landmark as would be seen by the participant performing the Cognitive Map Test, while viewing a video clip of movement throughout the environment. The correct landmark locations which participants would be attempting to report are shown in (**B**), and the four unique landmarks populating the environment are shown in (**C**).

The Cognitive Map Test is a well-known test adopted to measure the ability of the individuals to create and make use of a mental representation, or cognitive map, of an environment^54^. This test is set in a 5-by-5 rectangular grid of buildings, four of which are distinct landmarks, and the others identical, nondescript buildings (see Fig. 6). The environment has no global environmental cues (e.g., directional lighting) nor any distal landmarks (e.g., mountains), to force participants to create a mental representation solely from the landmarks experienced while navigating. At each trial, participants are first shown a 1-min video clip depicting first-person movement along the streets in the virtual city, pausing to view landmarks as they pass them. After each video clip, the participants were asked to indicate the positions of the four landmarks on an aerial view of the environment, with no restriction on the time allotted to respond. Trials continued until the correct spatial layout of landmarks was identified, or if 20 trials elapsed without a correct response, participants would be given a score of 21 trials. Performance is scored as the number of trials required to provide the correct layout, with a greater number of trials indicative of a poorer performance. All data reported from this task utilized the same layout, landmarks, and trial order.

### Data analyses

For the majority of measures we performed Bayesian analyses of covariance with heterogeneous covariate slopes in JASP (v0.12.2), with sex, DTD status, age, and all interactions modelled. A uniformly distributed prior was used as the initial likelihood for each of the 19 possible models (P(M) = 1/19), and the number of Markov chain Monte Carlo iterations was automatically determined by JASP. For each measure, effects across all models were inspected, and the model-averaged, i.e. unconditional, posterior estimates from evidenced DTD effects (i.e., BF_incl_ > 10, across matched models) are reported alongside their 99% credible intervals. Follow-up analyses of DTD-by-age interactions were performed using simple linear regression to report group-specific age slopes for interpretation. Nominal data were analyzed using chi-squared tests to detect differences in the proportions of a given variable between DTD and control groups (α = 0.01). All reaction time measures were ln-transformed before analysis. Due to the large number of statistical tests performed in the present dataset, we opted to use conservative statistical thresholds to reduce the rate of false positive results.

## Supplementary information

Supplementary Information 1.

Supplementary Information 2.

## Data Availability

Datasets and analyses are available from the corresponding author upon request.
