# Supplementary material for: Behavioural and cognitive mechanisms of Developmental Topographical Disorientation
Source: Sci Rep. 2020 Dec 1;10:20932. doi: 10.1038/s41598-020-77759-8 (PMC7708628; doi:10.1038/s41598-020-77759-8)
Supplement: Supplementary file 1 — Supplementary Information 1. [file 41598_2020_77759_MOESM1_ESM.docx]

**Behavioural and cognitive mechanisms of Developmental Topographical Disorientation**

Ford Burles and Giuseppe Iaria*

NeuroLab, Department of Psychology, University of Calgary, Calgary, Alberta, Canada.

*Correspondence to:

Giuseppe Iaria, PhD

Department of Psychology,

University of Calgary, AB, Canada

Email: giaria@ucalgary.ca

**Supplementary Materials 1**

**Table 1. Descriptive statistics and Bayes Factors of DTD and control samples by reported gender for questionnaires and interactive assessments**

See uploaded file.

**Supplementary Materials 2**

**Navigational Self-Assessment**

All items are scored on a 7-point Likert scale from 1 – Strongly Agree to 7 – Strongly Disagree.

1) I am very good at recognizing familiar faces

2) I have a poor ability for recognizing facial information such as expression or gender

3) My ability to recognize familiar objects such as cars, animals, or household objects, is very good

4) I am very good at recognizing familiar places

5) My ability to imagine familiar faces is very good

6) I am very good at imagining familiar objects

7) I have a poor ability to imagine familiar places

8) I am very good at discriminating between right and left

9) I use my GPS navigation system regularly when driving to familiar destinations

10) I use my GPS navigation system regularly when driving to unfamiliar destinations

**Supplementary Materials 3**

**DTD Family-Heritability Questionnaire**

1) Are you aware of your biological father experiencing orientation difficulties that arose in childhood? Yes / No / I don’t know

2) Is your biological father deceased? Yes / No / I don’t know

3) Are you aware of your biological mother experiencing orientation difficulties that arose in childhood? Yes / No / I don’t know

4) Is your biological mother deceased? Yes / No / I don’t know

5a) Number of known biological brothers you have:

5b) If 5a ≥ 1: Number of known biological brothers that may have orientation difficulties that arose in childhood:

6a) Number of known biological sisters you have:

6b) If 6a ≥ 1: Number of known biological sisters that may have orientation difficulties that arose in childhood:

7a) Number of known biological sons you have:

7b) If 7a ≥ 1: Number of known biological sons that may have orientation difficulties that arose in childhood:

8a) Number of known biological daughters you have:

8b) If 8a ≥ 1: Number of known biological daughters that may have orientation difficulties that arose in childhood:

9) Are you aware of any other biological relatives who may have had orientation difficulties that arose in childhood?

Supplementary Materials 4

Sample items and descriptions of the social and personality measures

The Positive and Negative Affect Schedule^58^ is a 20-item scale in which participants rate the extent to which they feel any given emotion (e.g. ‘Inspired’, ‘Nervous’, ‘Active’, ‘Guilty’, etc.) on a 5-point Likert scale ranging from ‘not at all’ to ‘extremely’. Ten items from this scale relate to negative emotions and form the ‘Negative Affect’ component, and the remaining ten refer to positive emotions and form the ‘Positive Affect’ component.

Lubben’s social network scale^59^ is a 12-item assessment of social engagement which includes items such as ‘How many of your friends do you see or hear from at least once a month?’ in which participants respond from options of 0, 1, 2, 3 to 4, 5 to 8, or 9 or more.

The Core Self-Evaluations Scale^60^ is a 12-item scale in which individuals indicate their agreement or disagreement with statements such as ‘Sometimes when I fail I feel worthless’ on a 5-point Likert scale ranging from ‘Strongly Agree’ to ‘Strongly Disagree’.

The New General Self-Efficacy Scale^61^ is an 8-item scale in which individuals indicate their agreement or disagreement with statements such as ‘I will be able to successfully overcome many challenges’ on a 5-point Likert scale ranging from ‘Strongly Agree’ to ‘Strongly Disagree’.

The Rosenberg Self-Esteem Scale^62^ is a 10-item scale in which individuals indicate their agreement or disagreement with statements such as ‘I am able to do things as well as most other people’ on a 5-point Likert scale ranging from ‘Strongly Agree’ to ‘Strongly Disagree’.

The Relationship Assessment Scale^63^ is a 7-item scale that was administered if participants indicated they were in a current romantic relationship. Participants responded to queries (*e.g.* ‘How well does your partner meet your needs?’) on a 5-point Likert scale ranging from ‘Poorly’ to ‘Extremely Well’.

The 5-item version of Levenson’s Locus of Control scale^64^ asks participants to rate the accuracy of statements (e.g. ‘Believe that unfortunate events occur because of bad luck’) relative to others their age and gender on a 5-point Likert scale ranging from ‘Very Inaccurate’ to ‘Very Accurate’.

The IPIP 60-item Extraversion and Neuroticism scales^65,66^ have participants rating the accuracy of statements (*e.g.* ‘Try to lead others’ and ‘Am afraid of many things’) on a 5-point Likert scale ranging from ‘Very Inaccurate’ to ‘Very Accurate’.
